# Surgical stabilization versus nonoperative treatment for flail and non-flail rib fracture patterns in patients with traumatic brain injury

**DOI:** 10.1007/s00068-022-01906-1

**Published:** 2022-02-22

**Authors:** Jonne T. H. Prins, Esther M. M. Van Lieshout, Francis Ali-Osman, Zachary M. Bauman, Eva-Corina Caragounis, Jeff Choi, D. Benjamin Christie, Peter A. Cole, William B. DeVoe, Andrew R. Doben, Evert A. Eriksson, Joseph D. Forrester, Douglas R. Fraser, Brendan Gontarz, Claire Hardman, Daniel G. Hyatt, Adam J. Kaye, Huan-Jang Ko, Kiara N. Leasia, Stuart Leon, Silvana F. Marasco, Allison G. McNickle, Timothy Nowack, Temi D. Ogunleye, Prakash Priya, Aaron P. Richman, Victoria Schlanser, Gregory R. Semon, Ying-Hao Su, Michael H. J. Verhofstad, Julie Whitis, Fredric M. Pieracci, Mathieu M. E. Wijffels

**Affiliations:** 1 Trauma Research Unit Department of Surgery, Erasmus MC, University Medical Center Rotterdam, P.O. Box 2040, 3000 CA Rotterdam, The Netherlands; 2 Department of Surgery, HonorHealth John C. Lincoln Medical Center, Phoenix, AZ 85020 USA; 3 Division of Trauma, Emergency General Surgery, Critical Care Surgery, Department of Surgery, University of Nebraska Medical Center, 983280 Nebraska Medical Center, Omaha, NE 68198-3280 USA; 4 Department of Surgery, Institute of Clinical Sciences, Sahlgrenska Academy, University of Gothenburg, Gothenburg, Sweden; 5 Section of Acute Care Surgery, Department of Surgery, Stanford University, Stanford, CA 94305 USA; 6 Department of Trauma Surgery/Critical Care, Mercer University School of Medicine, The Medical Center Navicent Health, Macon, GA 31201 USA; 7 HealthPartners Orthopedics and Sports Medicine, Bloomington, MN 55420 USA; 8 Department of Orthopaedic Surgery, University of Minnesota, Minneapolis, MN 55455 USA; 9 Department of Orthopaedic Surgery, Regions Hospital, Saint Paul, MN 55101 USA; 10 Department of Surgery, Riverside Methodist Hospital, Columbus, 43214 OH USA; 11 Department of Surgery, Saint Francis Hospital, Hartford, CT 06105 USA; 12 Division of Trauma and Critical Care, Department of Surgery, Medical University of South Carolina, Charleston, SC 29425 USA; 13 Department of Surgery, Kirk Kerkorian School of Medicine at UNLV, Las Vegas, NV 89102 USA; 14 Division of Trauma, Department of Surgery, Wright State University/Miami Valley Hospital, Dayton, OH 45409 USA; 15 Department of Surgery, Overland Park Regional Medical Center, Overland Park, KS 66215 USA; 16 Division of Trauma Surgery, Department of Surgery, National Taiwan University Hospital, Hsin-Chu Branch, Hsinchu, 30059 Taiwan; 17 Department of Surgery, Denver Health Medical Center, Denver, CO 80204 USA; 18 CJOB Department of Cardiothoracic Surgery, The Alfred, Melbourne, Australia; 19 Department of Surgery, Monash University, Clayton, VIC Australia; 20 Department of Surgery, Boston Medical Center, Boston University School of Medicine, Boston, MA USA; 21 Department of Trauma/Burn, John H Stroger Hospital of Cook County, Chicago, IL 60612 USA; 22 Department of Surgery, University of Texas Rio Grande Valley, Doctors Hospital at Renaissance, Edinburg, TX 78539 USA; 23 Department of Surgery, Denver Health Medical Center, University of Colorado School of Medicine, Denver, CO 80204 USA

**Keywords:** Surgical stabilization of rib fractures, Rib fracture, Flail chest, Traumatic brain injury, Thoracic trauma

## Abstract

**Purpose:**

Literature on outcomes after SSRF, stratified for rib fracture pattern is scarce in patients with moderate to severe traumatic brain injury (TBI; Glasgow Coma Scale ≤ 12). We hypothesized that SSRF is associated with improved outcomes as compared to nonoperative management without hampering neurological recovery in these patients.

**Methods:**

A post hoc subgroup analysis of the multicenter, retrospective CWIS-TBI study was performed in patients with TBI and stratified by having sustained a non-flail fracture pattern or flail chest between January 1, 2012 and July 31, 2019. The primary outcome was mechanical ventilation-free days and secondary outcomes were in-hospital outcomes. In multivariable analysis, outcomes were assessed, stratified for rib fracture pattern.

**Results:**

In total, 449 patients were analyzed. In patients with a non-flail fracture pattern, 25 of 228 (11.0%) underwent SSRF and in patients with a flail chest, 86 of 221 (38.9%). In multivariable analysis, ventilator-free days were similar in both treatment groups. For patients with a non-flail fracture pattern, the odds of pneumonia were significantly lower after SSRF (odds ratio 0.29; 95% CI 0.11–0.77; *p* = 0.013). In patients with a flail chest, the ICU LOS was significantly shorter in the SSRF group (beta, − 2.96 days; 95% CI − 5.70 to − 0.23; *p* = 0.034).

**Conclusion:**

In patients with TBI and a non-flail fracture pattern, SSRF was associated with a reduced pneumonia risk. In patients with TBI and a flail chest, a shorter ICU LOS was observed in the SSRF group. In both groups, SSRF was safe and did not hamper neurological recovery.

## Introduction

Traumatic brain injury (TBI) and thoracic trauma are the number one and two leading causes of trauma-related mortality annually, respectively [1, 2]. In the Intensive Care Unit (ICU), rib fractures and TBI are the most prevalent injuries and up to 25% of patients with multiple rib fractures have concomitant TBI [3, 4]. Both injuries are associated with prolonged mechanical ventilation requirement and ICU days, and combined they have been shown to increase the risk of pneumonia, which is a strong independent predictor of mortality after trauma [1, 3, 5].

Utilization of surgical stabilization of rib fractures (SSRF) has increased significantly over the last two decades [6–8]. In patients with a flail chest, SSRF has been associated with a reduced pneumonia rate, and shorter duration of mechanical ventilation and hospital and ICU length of stay (HLOS and ICU LOS) as compared to nonoperative management [9–13]. Studies specifically evaluating outcomes after SSRF in patients with a non-flail fracture pattern are scarce [14]. A recent randomized controlled trial indicated less pain at 2-week follow-up and fewer pleural space complications after SSRF in these patients [15]. Other injury characteristics for which SSRF have been recommended include ≥ 3 bi-cortically displaced rib fractures or a hemi-thorax volume loss of ≥ 30% [16]. The exact effect of SSRF in these populations remains uncertain however as these are often collectively evaluated with patients with a flail and non-flail fracture pattern [17].

The presence of TBI has been considered a relative contraindication for surgery, including SSRF and was often used as an exclusion criterion for rib fracture-related research [15, 18–20]. Recently however, the multicenter, retrospective Chest Wall Injury Society (CWIS)-TBI study reported SSRF to be safe in the presence of moderate to severe TBI (Glasgow Coma Scale [GCS] score ≤ 12) and associated with a reduced odds ratio of pneumonia and 30-day mortality [21]. This study was the first to specifically assess SSRF in the TBI population with rib fractures, but did not stratify by rib fracture pattern. As the established grounds for SSRF have expanded, a small number of studies have assessed the flail chest and non-flail fracture pattern separately due to their injury-related dissimilarities [14, 22].

Therefore, the aim of this study was to evaluate the effect of SSRF versus nonoperative management in patients with TBI and either a flail chest or non-flail fracture pattern on ventilator-free days. Secondary aims were to assess in-hospital outcomes, such as pneumonia rate, motor neurological status, HLOS, ICU LOS, and mortality. We hypothesized that SSRF is associated with improved outcomes including more ventilator-free days, shorter ICU LOS, and a lower pneumonia rate, as compared to nonoperative management without hampering neurological recovery in patients with both flail and non-flail rib fracture patterns.

## Methods

### Design and participants

**Fig. 1:**
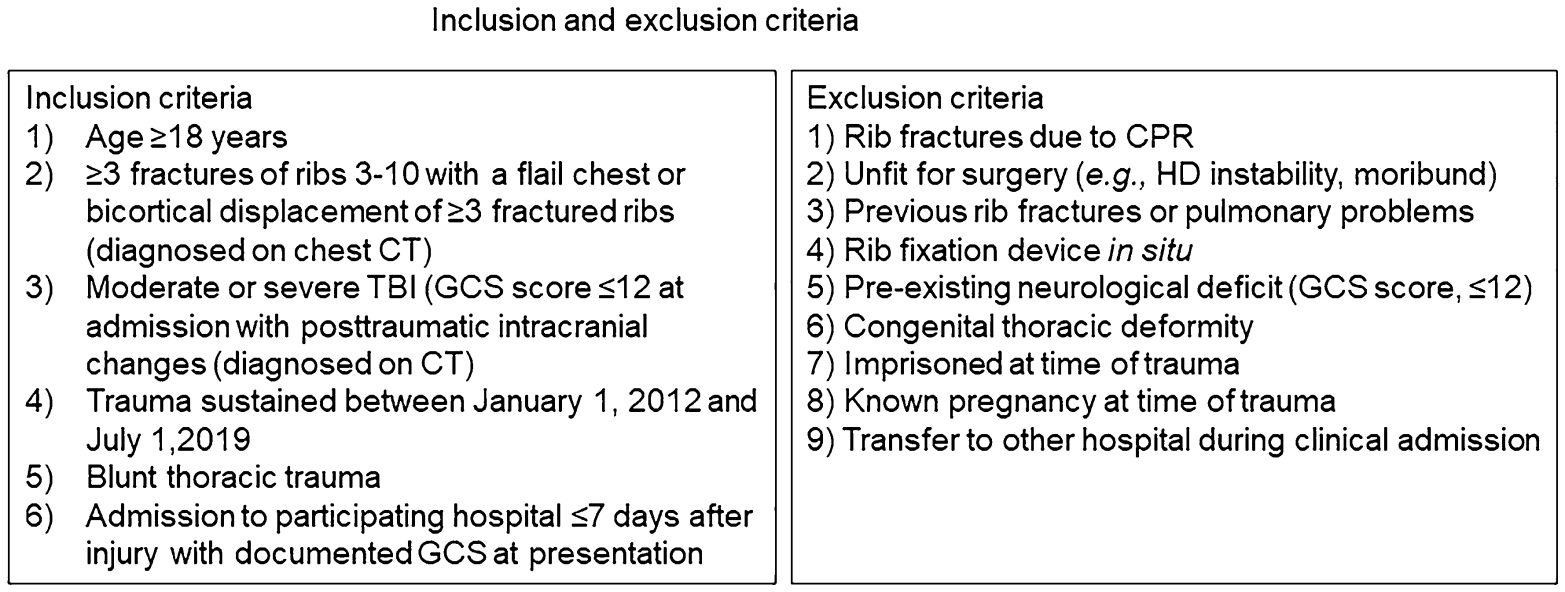
Study inclusion and exclusion criteria. *CPR* cardiopulmonary resuscitation, *CT* computed tomography, *GCS* Glasgow Coma Scale, *HD* hemodynamic, *TBI* traumatic brain injury

This CWIS-TBI study was a multicenter, retrospective cohort study involving 19 trauma centers conducted through the Chest Wall Injury Society (http://www.cwisociety.org) [21]. The study was approved by each center’s local medical research ethics committee or institutional review board and informed consent was exempted. Eligible patients were identified through the hospitals’ electronic medical record and by searching their trauma registry for admitted patients with a registered Abbreviated Injury Scale (AIS) for rib or sternal fractures in combination with an AIS ≥ 3 of the head. Figure 1 lists the inclusion and exclusion criteria. Patients were stratified by having sustained a flail chest or non-flail fracture pattern. A flail chest was defined as having sustained ≥ 3 bi-cortical consecutive ribs fractured in two or more locations on chest computed tomography (CT; radiographic flail segment) or ≥ 3 ribs fractured with a paradoxical chest wall respiratory motion (physiologic flail chest). A non-flail fracture pattern was defined as the absence of a radiographic on chest CT or physiologic flail chest.

### Data collection and outcome measures

The primary outcome measure was the number of ventilator-free days during primary hospital admission, defined as the number of days the patient breathed without assisted (non)-invasive ventilation. Secondary outcome measures were ICU LOS, HLOS, the occurrence of thoracic complications (i.e., pneumonia within 30 days as defined according to the Centers for Disease Control and Prevention (CDC) guidelines [23], pleural empyema within 30 days as diagnosed on CT scan and/or pus evacuation [24]), and SSRF-related complications (i.e., superficial and deep wound infection, post-operative bleeding, implant failure requiring removal, and perioperative intracranial pressure increase requiring [non]invasive intervention), neurological outcome (rate of and time to motor GCS [mGCS] score = 6 achieved), and < 30 days and in-hospital mortality.

In addition to the outcome measures, patient characteristics and injury-related variables were collected. The TBI severity at hospital admission was defined as moderate (GCS score, 9–12) or severe (GCS score, ≤ 8). Intracranial hypertension was defined as an intracranial pressure (ICP) of > 20 mmHg. Also, treatment- and outcome-related variables were collected. Therapy for reducing ICP consisted of having received or undergone ≥ 1 of the following: mannitol, hypertonic saline, pentobarbital, ventriculostomy, craniotomy, or placement of a subdural evacuation port system.

### Statistical analysis

Data were analyzed using the Statistical Package for the Social Sciences (SPSS) version 25 or higher (SPSS, Chicago, Ill., USA). Normality of continuous variables was tested with the Shapiro–Wilk test, and homogeneity of variances was tested using the Levene’s test. A *p *value lower than 0.05 was considered statistically significant and all tests were two-sided. Descriptive analysis was performed to report the data for the entire flail chest and non-flail fracture pattern population and for the treatment groups. For continuous data, the median and percentiles (non-parametric data) were reported. Statistical significance of differences between treatment groups was assessed using Mann–Whitney *U* test (non-parametric data). For categorical data, numbers and frequencies are reported per treatment group and compared using Chi-squared or Fisher’s exact test, as applicable.

In multivariable analysis, a regression model was developed to control for potential confounders, as described in the main study manuscript [21]. The final regression model for the non-flail fracture pattern group consisted of the covariates number of fractured ribs, chest tube requirement, and intracranial hypertension presence. The model for the flail chest group consisted of BMI, COPD, number of fractured ribs, chest tube requirement, and intracranial hypertension presence. Given the multicenter design of the study, participating center was also considered as a confounder. Study center was however not included in the final model as it did not statistically correlate with outcomes. The final crude regression model included the outcome measure as the dependent variable, and SSRF as covariate. In the adjusted analysis, the covariates mentioned above were added as covariates. For binary regression analysis, the OR for SSRF over nonoperative treatment is reported with 95% confidence interval (CI) and *p *values. For linear regression analysis, the beta value with 95% CI and *p* value is reported.

## Results

**Fig. 2:**
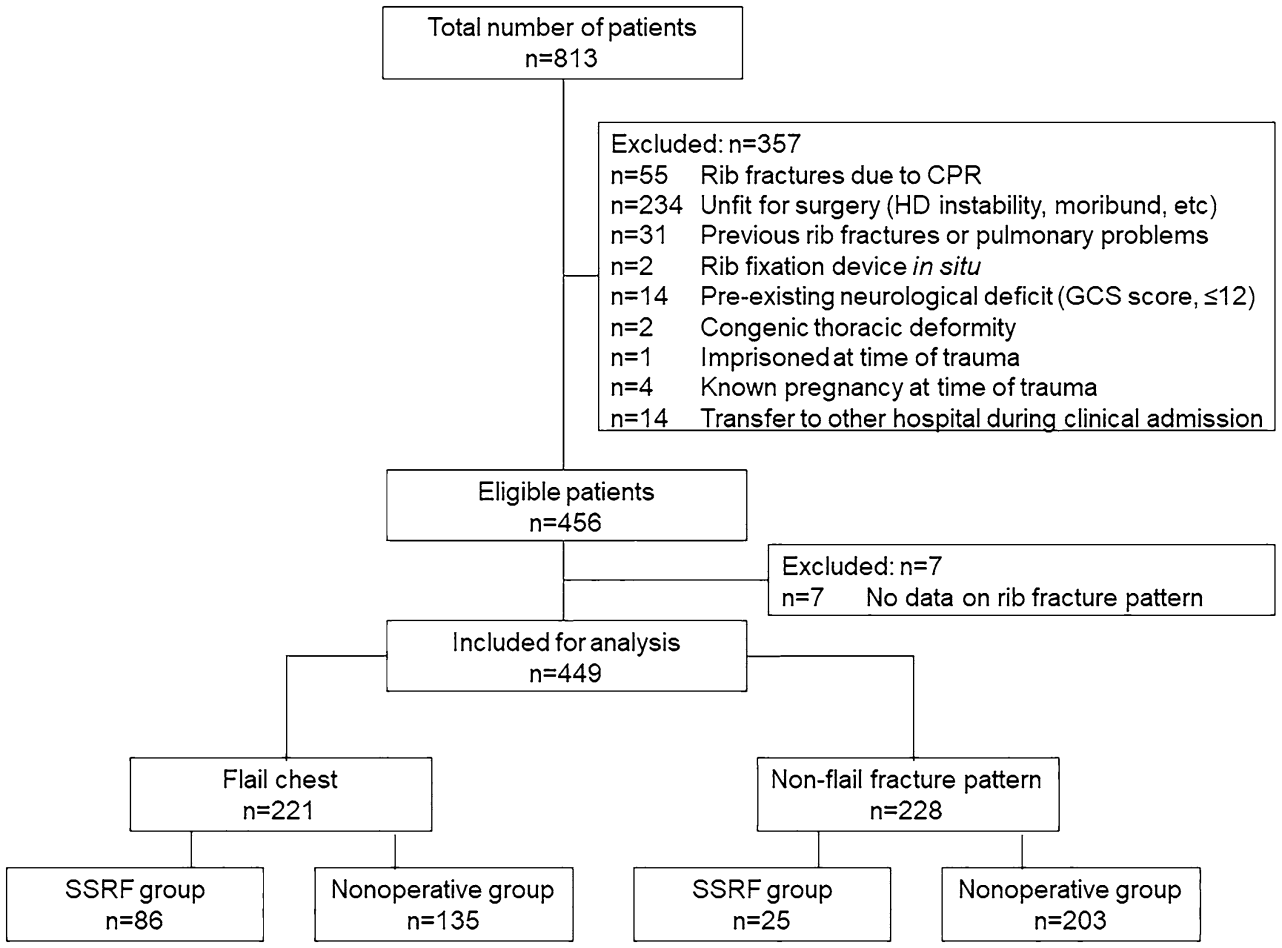
Study flow chart. *CPR* cardiopulmonary resuscitation, *GCS* Glasgow Coma Scale, *HD* hemodynamic, *SSRF* surgical stabilization of rib fractures

In total, 449 (55.2%) patients with multiple rib fractures and TBI were included (Fig. 2). For each study center, the number of included patients with multiple rib fractures and TBI ranged from 2 to 65. The percentage of these patients who underwent SSRF ranged from 0 to 67%.

### Patients with a non-flail fracture pattern

In total, 228 patients had a non-flail fracture pattern, of whom 25 (11.0%) underwent SSRF (Table 1). Operatively treated patients had a higher number of fractured ribs (8, *P*_25_–*P*_75_ 7–12, vs. 6, *P*_25_–*P*_75_ 5–9; *p* = 0.009). In addition, these patients had severe TBI less frequently (*n* = 14, 56% vs. *n* = 163, 80.3%; *p* = 0.010) than nonoperatively treated patients. Other patient and injury characteristics, such as the presence of intracranial hypertension after trauma, were similar. Regarding treatment characteristics, patients who underwent SSRF more often required a chest tube at admission (*n* = 22, 88% vs. *n* = 121, 59.6%; *p* = 0.004) and less often ICP reducing therapy (*n* = 4, 16% vs. *n* = 81, 39.9%; *p* = 0.027; Table 1). SSRF was performed at a median of 2 days (*P*_25_–*P*_75_ 1–6) after trauma during which a median of 5 ribs (*P*_25_–*P*_75_ 4–6) were repaired, resulting in a ratio of ribs repaired to fractured of 0.5 (*P*_25_–*P*_75_ 0.4–0.8). Two patients developed a wound infection (8%) following SSRF, of which one required implant removal. There were no perioperative neurological complications, post-operative bleeding, or implant failure during hospitalization in the SSRF group.

**Table 1:** Patient, injury, and treatment characteristics and in-hospital outcomes in patients with a non-flail rib fracture pattern and moderate to severe traumatic brain injury who underwent surgical stabilization of rib fractures (SSRF) or nonoperative management

	All (*n* = 228)		SSRF (*n* = 25)		Nonoperative (*n* = 203)		*p *value
	*N*^a^		*N*^a^		*N*^a^		
*Patient characteristics*							
Age (years)	228	49 (35–63	25	50 (32–62)	203	49 (35–64)	0.797
BMI (kg/m^2^)	194	25.8 (23.5–29.4)	22	27.2 (24.3–29.7)	172	25.9 (23.1–29.4)	0.371
Sex (male)	228	174 (76.3%)	25	17 (68%)	203	157 (77.3%)	0.321
Smoking	160	71 (44.4%)	21	10 (48%)	139	61 (43.9%)	0.816
COPD	228	10 (4.4%)	25	0 (0%)	203	10 (4.9%)	0.607
Diabetes mellitus	228	25 (11.0%)	25	3 (12%)	203	22 (10.8%)	0.743
*Injury characteristics*							
Fractured ribs (*N*)	228	7 (5–9)	25	8 (7–12)	203	6 (5–9)	**0.009**
Bilateral rib fractures	228	83 (36.4%)	25	9 (36%)	203	74 (36.5%)	1.000
Fracture in every rib region	194	34 (17.5%)	20	5 (25%)	174	29 (16.7%)	0.356
≥ 100% displacement of ≥ 3 ribs	223	159 (71.3%)	24	21 (88%)	199	138 (69.3%)	0.092
Pneumothorax	228	153 (67.1%)	25	20 (80%)	203	133 (65.5%)	0.179
Hemothorax	228	101 (44.5%)	25	13 (52%)	203	88 (43.6%)	0.523
Pulmonary contusion	228	165 (73.0%)	25	21 (84%)	203	144 (71.6%)	0.237
ISS	228	29 (25–38)	25	29 (25–36)	203	33 (26–41)	0.502
Epidural hematoma	228	23 (10.1%)	25	3 (12%)	203	20 (9.9%)	0.725
Subdural hematoma	228	127 (55.7%)	25	9 (36%)	203	118 (58.1%)	0.053
Subarachnoid hemorrhage	228	154 (67.5%)	25	15 (60%)	203	139 (68.5%)	0.497
Diffuse axonal injury	228	44 (19.3%)	25	5 (20%)	203	39 (19.2%)	1.000
Intra-parenchymal hemorrhage	228	76 (33.3%)	25	12 (48%)	203	64 (31.5%)	0.117
Intraventricular hemorrhage	228	16 (7.0%)	25	1 (4%)	203	15 (7.4%)	1.000
Brain contusion	228	18 (7.9%)	25	4 (16%)	203	14 (6.9%)	0.119
Intracranial hypertension	228	50 (22.4%)	25	2 (8%)	203	48 (24.2%)	0.077
Severe TBI (GCS ≤ 8)	228	177 (77.6%)	25	14 (56%)	203	163 (80.3%)	**0.010**
*Treatment characteristics*							
Chest tube required	228	134 (62.7%)	25	22 (88%)	203	121 (59.6%)	**0.004**
ICP reducing therapy required	228	87 (38.2%)	25	4 (16%)	203	83 (40.9%)	**0.016**
Tracheostomy required	228	88 (38.6%)	25	10 (40%)	203	78 (38.4%)	1.000
Additional surgeries required							
Emergency thoracotomy	228	6 (2.6%)	25	1 (4%)	203	5 (2.5%)	0.506
Emergency laparotomy	228	25 (11.0%)	25	2 (8%)	203	23 (11.3%)	1.000
Pelvic surgery	228	24 (10.5%)	25	1 (4%)	203	23 (11.3%)	0.487
Long bone surgery	228	48 (21.1%)	25	4 (16%)	203	44 (21.7%)	0.612
Spine surgery	228	29 (12.7%)	25	2 (8%)	203	27 (13.3%)	0.750
*Outcome characteristics*							
Mechanical ventilation (days)	228	10 (4–18)	25	8 (4–19)	203	10 (4–18)	0.802
Ventilator-free days (days)	228	10 (2–21)	25	9 (7–17)	203	11 (1–23)	0.815
Motor GCS score recovery to 6	214	172 (80.4%)	23	23 (100%)	191	149 (78.0%)	**0.010**
Time to motor GCS 6 (days)	162	3 (1–11)	22	2 (1–6)	140	4 (1–14)	**0.045**
Pneumonia	228	102 (44.7%)	25	6 (24%)	203	96 (47.3%)	**0.033**
Pleural empyema	228	4 (1.8%)	25	0 (0%)	203	4 (2%)	1.000
ICU LOS	228	13 (6–21)	25	12 (7–20)	203	13 (6–21)	0.921
HLOS	228	21 (13–39)	25	21 (14–28)	203	21 (13–40)	0.681
30-day mortality	228	36 (15.8%)	25	0 (0%)	203	36 (17.7%)	**0.018**
In-hospital mortality	228	40 (17.5%)	25	1 (4%)	203	39 (19.2%)	0.090
Mortality cause							
Traumatic brain injury	31	13 (42%)	1	0 (0%)	30	13 (43%)	0.366
Pulmonary origin	31	5 (16%)	1	0 (0%)	30	5 (17%)	
Septic shock	31	6 (19%)	1	0 (0%)	30	6 (20%)	
Hemorrhagic shock	31	0 (0%)	1	0 (0%)	30	0 (0%)	
Cardiac origin	31	1 (3%)	1	0 (0%)	30	1 (3%)	
Withdrawal of care	31	6 (19%)	1	1 (17%)	30	5 (17%)	

Data are shown as median (*P*_25_–*P*_75_) or as *N* (%)

*BMI* Body Mass Index, *COPD* chronic obstructive pulmonary disease, *GCS* Glasgow Coma Scale, *HLOS* hospital length of stay, *ICP* intracranial pressure, *ICU LOS* intensive care unit length of stay, *ISS* injury severity score, *SSRF* surgical stabilization of rib fractures, *TBI* traumatic brain injury

Bold and underlined *p *values are considered statistically significant

^a^Provides the exact number of patients for whom data were available

In univariate analysis, ventilator-free days were similar in both the operative and nonoperative group (Table 1). The SSRF group had a lower rate of pneumonia (*n* = 6, 24% vs. *n* = 96, 47.3%; *p* = 0.033) and 30-day mortality (*n* = 0, 0% vs. *n* = 36, 17.7%; *p* = 0.018). The SSRF group also had a higher rate of mGCS recovery to 6 (*n* = 23, 100% vs. *n* = 149, 78.0%; *p* = 0.010) which was achieved in fewer days (2 days, *P*_25_–*P*_75_ 1–6 vs. 4 days, *P*_25_–*P*_75_ 1–14; *p* = 0.045).

In multivariable adjusted analysis, ventilator-free days did not differ between the treatment groups (Table 2). Odds of developing pneumonia were significantly lower in patients who underwent SSRF (OR 0.29; 95% CI 0.11–0.77; *p* = 0.013). Other outcomes, including mortality, were similar across the treatment groups.

**Table 2:** Multivariable in-hospital outcomes of surgical stabilization of rib fractures versus nonoperative treatment in patients with a non-flail rib fracture pattern and moderate to severe traumatic brain injury

Outcome	Multivariable analysis					
	Crude analysis			Adjusted analysis		
	*N*^a^	Beta or OR (95% CI)	*p *value	*N*^a^	Beta or OR (95% CI)	*p *value
Ventilator-free days	228	− 4.09 (− 12.27 to 4.10)	0.326	228	− 5.91 (− 14.39 to 2.58)	0.171
ICU LOS	228	− 1.24 (− 6.13 to 3.65)	0.618	228	− 2.85 (− 7.82 to 2.12)	0.260
HLOS	228	− 5.32 (− 14.96 to 4.31)	0.278	228	− 8.62 (− 18.55 to 1.31)	0.089
Pneumonia	228	0.35 (0.14 to 0.92)	**0.033**	228	0.29 (0.11 to 0.77)	**0.013**
Motor GCS score recovery to 6	214	ND	ND	214	ND	ND
In-hospital mortality	228	0.18 (0.02 to 1.34)	0.093	228	0.24 (0.03 to 1.90)	0.176
30-day mortality	228	ND	ND	228	ND	ND

The multivariable analysis shows the effect of SSRF over nonoperative treatment. In the adjusted analysis, the number of fractured ribs, chest tube requirement, and presence of intracranial hypertension were entered as covariate

*CI* confidence interval, *HLOS* hospital length of stay, *ICU LOS* intensive care unit length of stay, *mGCS* motor Glasgow Coma Scale, *ND* not determined, *OR* odds ratio

Data are shown as odds ratio (OR; categorical outcome) or beta (continuous outcome) with 95% confidence interval. Bold and underlined p values are considered statistically significant. and underlined

^a^Provides the exact number of patients for whom data were available

### Patients with a flail chest

In total, 221 patients had a flail chest of whom 86 (38.9%) underwent SSRF (Table 3). These patients more often had COPD than the nonoperative group (*n* = 11, 13% vs. *n* = 12, 8.9%; *p* = 0.016). Other patient and thoracic injury characteristics were similar across groups. Following injury, the SSRF group had lower rates of intracranial hypertension (*n* = 10, 12% vs. *n* = 38, 29.0%; *p* = 0.003), severe TBI (*n* = 62, 72% vs. *n* = 116, 85.9%; *p* = 0.015) and less often required ICP reducing therapy (*n* = 21, 24% vs. *n* = 59, 43.7%; *p* = 0.004). Also, patients who underwent SSRF more often required a chest tube at admission (*n* = 77, 90% vs. *n* = 103, 76.3%; *p* = 0.014). Patients underwent SSRF at a median of 3 days (*P*_25_–*P*_75_ 2–5) during which a median of 4 ribs (*P*_25_–*P*_75_ 3–5) were repaired, resulting in a ratio of ribs repaired to fractured of 0.5 (*P*_25_–*P*_75_ 0.4–0.6). Two SSRF-related complications occurred (2.3%): one patient developed increased intraoperative intracranial pressure requiring mannitol and reverse Trendelenburg positioning after which the SSRF was completed, and one mechanical implant failure requiring implant removal during the hospitalization.

**Table 3:** Patient, injury, and treatment characteristics and in-hospital outcomes in patients with a flail chest and moderate to severe traumatic brain injury who underwent surgical stabilization of rib fractures (SSRF) or nonoperative management

	All (*n* = 221)		SSRF (*n* = 86)		Nonoperative (*n* = 135)		*p *value
	*N*^a^		*N*^a^		*N*^a^		
*Patient characteristics*							
Age (years)	221	51 (40–62)	86	49 (38–60)	135	51 (42–62)	0.508
BMI (kg/m^2^)	189	27.1 (23.9–31.3)	78	27.8 (24.6–32.6)	120	26.3 (23.5–30.0)	0.057
Sex (male)	220	168 (76.4%)	85	63 (74%)	135	105 (77.8%)	0.625
Smoking	163	59 (36.2%)	101	28 (45%)	62	31 (30.7%)	0.067
COPD	221	16 (7.2%)	86	11 (13%)	135	5 (3.7%)	**0.016**
Diabetes mellitus	221	23 (10.4%)	86	11 (13%)	135	12 (8.9%)	0.373
*Injury characteristics*							
Fractured ribs (*N*)	221	9 (8–12)	86	9 (8–12)	135	9 (7–12)	0.855
Bilateral rib fractures	221	100 (45.2%)	86	35 (41%)	135	65 (48.1%)	0.332
Fracture in every rib region	207	104 (50.2%)	80	43 (54%)	127	61 (48.0%)	0.476
≥ 100% displacement of ≥ 3 ribs	214	138 (64.5%)	85	60 (71%)	129	78 (60.5%)	0.146
Pneumothorax	221	186 (84.2%)	86	74 (86%)	135	112 (83.0%)	0.577
Hemothorax	220	140 (63.6%)	85	54 (64%)	135	86 (63.7%)	1.000
Pulmonary contusion	219	165 (75.3%)	86	64 (74%)	133	101 (75.9%)	0.873
ISS	221	34 (29–44)	86	34 (29–43)	135	36 (29–45)	0.235
Epidural hematoma	221	15 (6.8%)	86	3 (3%)	135	12 (8.9%)	0.171
Subdural hematoma	221	110 (49.8%)	86	32 (37%)	135	78 (57.8%)	**0.004**
Subarachnoid hemorrhage	221	141 (63.8%)	86	45 (52%)	135	96 (71.1%)	**0.006**
Diffuse axonal injury	221	43 (19.5%)	86	16 (19%)	135	27 (20.0%)	0.863
Intra-parenchymal hemorrhage	221	55 (24.9%)	86	22 (26%)	135	33 (24.4%)	0.874
Intraventricular hemorrhage	221	24 (10.9%)	86	4 (5%)	135	20 (14.8%)	**0.025**
Brain contusion	221	50 (22.6%)	86	22 (26%)	135	28 (20.7%)	0.414
Intracranial hypertension	221	48 (22.1%)	86	10 (12%)	135	38 (29.0%)	**0.003**
Severe TBI (GCS ≤ 8)	221	178 (80.5%)	86	62 (72%)	135	116 (85.9%)	**0.015**
*Treatment characteristics*							
Chest tube required	221	180 (81.4%)	86	77 (90%)	135	103 (76.3%)	**0.014**
ICP reducing therapy required	221	81 (36.7%)	86	22 (26%)	135	59 (43.7%)	**0.007**
Tracheostomy required	221	81 (36.7%)	86	25 (29%)	135	56 (41.5%)	0.065
Additional surgeries required							
Emergency thoracotomy	221	12 (5.4%)	86	7 (8%)	135	5 (3.7%)	0.223
Emergency laparotomy	221	29 (13.1%)	86	9 (11%)	135	20 (14.8%)	0.417
Pelvic surgery	221	22 (10.0%)	86	10 (12%)	135	12 (8.9%)	0.501
Long bone surgery	221	58 (26.2%)	86	29 (34%)	135	29 (21.5%)	0.059
Spine surgery	221	17 (7.7%)	86	4 (5%)	135	13 (9.6%)	0.205
*Outcome characteristics*							
Mechanical ventilation (days)	221	10 (5–18)	86	9 (5–14)	135	11 (5–21)	**0.040**
Ventilator-free days (days)	221	11 (4–21)	86	13 (8–20)	135	9 (1–21)	**0.034**
Motor GCS score recovery to 6	206	161 (78.2%)	80	73 (91%)	126	88 (69.8%)	** < 0.001**
Time to motor GCS 6 (days)	144	4 (2–11)	69	4 (1–9)	75	5 (2–14)	0.075
Pneumonia	221	98 (44.3%)	86	32 (37%)	135	66 (48.9%)	0.097
Pleural empyema	221	2 (0.9%)	86	1 (1%)	135	1 (0.7%)	1.000
ICU LOS	221	14 (7–21)	86	12 (7–17)	135	15 (7–23)	0.066
HLOS	221	22 (14–34)	86	22 (16–33)	135	23 (11–35)	0.914
30-day mortality	221	33 (14.9%)	86	7 (8%)	135	26 (19.3%)	**0.032**
In-hospital mortality	221	34 (15.4%)	86	7 (8%)	135	27 (20.0%)	**0.021**
Mortality cause							
Traumatic brain injury	28	13 (46%)	7	2 (29%)	21	11 (52%)	0.191
Pulmonary origin	28	6 (21%)	7	1 (14%)	21	5 (24%)	
Septic shock	28	3 (11%)	7	2 (29%)	21	1 (5%)	
Hemorrhagic shock	28	1 (4%)	7	1 (14%)	21	0 (0%)	
Cardiac origin	28	3 (11%)	7	1 (14%)	21	2 (10%)	
Withdrawal of care	28	1 (4%)	7	0 (0%)	21	2 (10%)	

Data are shown as median (*P*_25_–*P*_75_) or as *N* (%)

*BMI* Body Mass Index, *COPD* Chronic Obstructive Pulmonary Disease, *HLOS* hospital length of stay, *GCS* Glasgow Coma Scale, *ICP* intracranial pressure, *ICU LOS* intensive care unit length of stay, *ISS* injury severity score, *SSRF* surgical stabilization of rib fractures, *TBI* traumatic brain injury

Bold and underlined *p *values are considered statistically significant

^a^Provides the exact number of patients for whom data were available

In univariate analysis, SSRF patients had more ventilator-free days than nonoperatively treated patients (13 days, *P*_25_–*P*_75_ 8–20 vs. 9 days, *P*_25_–*P*_75_ 1–21; *p* = 0.034; Table 3). The SSRF had lower 30-day (*n* = 7, 8% vs. *n* = 26, 19.3%; *p* = 0.032) and in-hospital mortality than the nonoperative group (*n* = 7, 8% vs. *n* = 27, 20.0%; *p* = 0.021). Patients who underwent SSRF had a higher rate of mGCS recovery to 6 (*n* = 73, 91% vs. *n* = 88, 69.8%; *p* < 0.001).

In multivariable adjusted analysis, ventilator-free days did not differ between treatment groups (Table 4). The SSRF group showed a significantly shorter ICU LOS (beta − 2.96 days; 95% CI − 5.70 to − 0.23; *p* = 0.034) and higher odds of mGCS recovery to 6 (OR 3.98; 95% CI 1.40–11.33; *p* = 0.010). Other outcomes, including mortality, were similar in both groups.

**Table 4:** Multivariable in-hospital outcomes of surgical stabilization of rib fractures versus nonoperative treatment in patients with a flail chest and moderate to severe traumatic brain injury

Outcome	Multivariable analysis					
	Crude analysis			Adjusted analysis		
	*N*^a^	Beta or OR (95% CI)	*p* value	N^a^	Beta or OR (95% CI)	*p* value
Ventilator-free days	221	1.25 (− 2.85 to 5.35)	0.547	221	− 0.28 (− 4.91 to 4.35)	0.905
ICU LOS	221	− 2.72 (− 5.21 to − 0.23)	**0.033**	221	− 2.96 (− 5.70 to − 0.23)	**0.034**
HLOS	221	− 1.76 (− 6.82 to 3.30)	0.494	221	− 3.36 (− 8.97 to 2.26)	0.240
Pneumonia	221	0.62 (0.36 to 1.08)	0.089	221	0.75 (0.39 to 1.43)	0.382
Motor GCS score recovery to 6	206	4.50 (1.90 to 10.68)	**0.001**	206	3.98 (1.40 to 11.33)	**0.010**
In-hospital mortality	221	0.35 (0.15 to 0.86)	**0.021**	221	0.39 (0.12 to 1.26)	0.114
30-day mortality	221	0.37 (0.15 to 0.90)	**0.028**	221	0.40 (0.12 to 1.29)	0.126

The multivariable analysis shows the effect of SSRF over nonoperative treatment. In the adjusted analysis, BMI, COPD, the number of fractured ribs, chest tube requirement, and presence of intracranial hypertension were entered as covariate

*BMI* body mass index, *CI* confidence interval, *COPD* Chronic Obstructive Pulmonary Disease, *HLOS* hospital length of stay, *ICU LOS* intensive care unit length of stay, *mGCS* motor Glasgow Coma Scale, *OR* odds ratio

Data are shown as odds ratio (OR; categorical outcome) or beta (continuous outcome) with 95% confidence interval. Bold and underlined *p *values are considered statistically significant.

^a^Provides the exact number of patients for whom data were available

## Discussion

This study investigated the effect of SSRF versus nonoperative management on in-hospital outcomes in patients with a flail or non-flail fracture pattern and concomitant TBI. For both types of rib fracture patterns, no beneficial effect of SSRF on the primary outcome of ventilator-free days was demonstrated. In patients with a flail chest, a 3-day decrease in ICU LOS was observed in patients who underwent SSRF. In patients with a non-flail fracture pattern, SSRF was associated with three times lower odds of pneumonia. In both rib fracture groups, SSRF was safe with a low complication rate and no pre- or postoperative neurological deterioration.

Patients with multiple rib fractures and TBI are often not considered candidates for SSRF, regardless of pulmonary abnormalities [12, 13]. This reason is likely multifactorial: the perioperative setting might cause increased intracranial pressure and patients with TBI are often expected to have lengthy mechanical ventilation requirement and ICU LOS, making it difficult to distill an effect of the severe rib fractures and SSRF on in-hospital outcomes. This dogma was challenged by the CWIS-TBI study, which showed that SSRF did not impair neurological recovery, had a low perioperative risk, and was associated with a lower risk of pneumonia and mortality [21]. As follow-up to this study, CWIS-TBI data were used to evaluate whether more specific rib fracture patterns benefit from SSRF. Patients with a non-flail fracture pattern who underwent SSRF had relatively similar thoracic injuries as compared to the nonoperative group. Patients with a flail chest had more severe thoracic injuries in the SSRF group and more severe brain injuries in the nonoperative group. This finding might provide reflection of the surgeon’s decision-making who considers TBI a contraindication for SSRF, and subsequently is more likely to offer SSRF to patients with the more severe rib fracture patterns and less severe TBI characteristics or improved neurologic prognosis. For both rib fracture pattern groups, the current study indicates that SSRF is safe and might be of benefit in these patients.

In patients with a flail chest, SSRF has previously been associated with decreased ICU LOS, as compared to nonoperative treatment [18, 20, 25, 26]. Several of these studies however, including two randomized controlled trials, specifically excluded patients with TBI [5, 18, 20]. In the current study, a shorter ICU LOS was observed in the SSRF group of patients with a flail chest, and SSRF was safe without signs of peri-procedural neurologic deterioration in the patient with TBI. This ICU LOS decrease did not result in shorter HLOS or increased ventilator-free days on multivariable analysis. This might be due to for example the effect of TBI extent or another unaccounted confounder which impacted ventilator-free days more strongly than chest wall injury severity or SSRF. This is supported by the increased ventilator-free days on univariate analysis for the SSRF group which was similar on multivariable analysis after correcting for intracranial hypertension presence. Also, with no data on mechanical ventilation mode, SSRF might have improved respiratory mechanics, assisted in stabilizing the patient, and allowed for a quicker wean and more rapid discharge from the ICU after complete ventilation liberation. A shorter ICU stay is also beneficial for the cost-effectiveness as SSRF has been shown to be economically more beneficial regarding hospital charges [26, 27].

Literature on the effect of SSRF versus nonoperative treatment in patients with a non-flail fracture pattern is scarce [14]. Only three studies have assessed the outcome pneumonia and either excluded patients with TBI or did not provide insight in patient selection [15, 28, 29]. This study is the first to specifically assess pneumonia rates following SSRF or nonoperative treatment in patients with a non-flail fracture pattern and TBI. On multivariable analysis, SSRF was associated with three times lower odds for developing pneumonia. Interestingly, this lower risk did not appear to have clinical consequences in terms of shorter hospital or ICU stay or increased ventilator-free days. It does highlight that besides TBI, chest wall injury plays a role in developing pneumonia and SSRF might be beneficial in reducing this risk.

Furthermore, as has been corroborated by the previous CWIS-TBI study, SSRF is a safe procedure in patients with TBI, also when specifically evaluated in chest wall injury subgroups. With high rates of mGCS score recovery to 6 and a low complication rate, SSRF and the consequent perioperative setting is safe and does not hamper neurological recovery. This is of importance as early SSRF (≤ 48–72 h after trauma) is associated with shorter HLOS, ICU LOS, mechanical ventilation duration, and lower rates of pneumonia [30–32]. With a median time from trauma to SSRF of 2 and 3 days in patients with a non-flail fracture pattern and a flail chest, respectively, this benefit of early SSRF might already be present. The optimal timing of SSRF in this population requires further evaluation. The benefit of early SSRF and the demonstrated safe perioperative SSRF setting might assist surgeons in decision-making in the acute setting when neurological prognosis is often unsure.

The results of this study should be interpreted acknowledging several limitations. First, the inclusion criterion of TBI through using a single GCS score at admission has known limitations (e.g., in intoxicated patients) and might be of less clinical significance than ongoing GCS score assessment or the GCS score at the day of SSRF. To minimize the impact of this limitation, the presence of intracranial injuries on brain CT was required. In addition, patients were identified for having a head AIS of ≥ 3 besides rib fractures, thus excluding patients with minor TBI with a lowered GCS. Also, the GCS score is the most commonly used parameter to assess TBI severity and is readily available in the acute setting in contrast to the AIS [33, 34]. Furthermore, the regression model corrected for TBI severity through the variable intracranial hypertension which was more strongly associated with outcomes than individual intracranial injuries. Future research should prospectively evaluate (acute and long-term) outcomes in the patient with TBI and use standardized treatment protocols across centers, consider ongoing GCS scores or on the day of SSRF instead of at admission, whether intracranial hypertension might be a SSRF contraindication instead of the general umbrella title TBI, and TBI improvement post-SSRF through CT scan instead of mGCS.

Second, the observational non-randomized study design might have introduced selection bias. Patients who are selected for SSRF often have more severe thoracic injuries but are also younger with less comorbidities than those treated nonoperatively, requiring adjusting for when assessing outcomes [35, 36]. In the current study, the treatment groups were relatively similar regarding thoracic injury severity but had significant dissimilarities in the severity of TBI and rate of associated intracranial injuries, being higher in the nonoperative group. Previously, recommendation of SSRF has been shown to be significantly impacted by TBI presence and degree; the more severe TBI, the less likely SSRF was recommended [37]. The prognosis assessment in patients with TBI remains difficult and a standardized treatment protocol regarding SSRF in this population is lacking [12, 38]. This might have resulted in SSRF being performed in patients with a better neurological status or those who were expected to have improved outcomes in terms of (neurological) recovery and during hospitalization, confounding observed outcomes which might subsequently be more strongly affected by the effect of the associated injuries than the treatment effect. To mitigate this effect, multivariable analysis was performed adjusting for intracranial hypertension. However, the extent to which the individual intracranial injuries or other uncaptured confounders might have affected outcomes or (not) being selected for SSRF remains unknown.

Third, the multicenter design might have impacted outcomes as both the numbers of included patients and rates of SSRF performed varied significantly between centers. Also, since there was no standardized (non)operative treatment protocol, heterogeneity of managing rib fractures across centers or potential confounding of within-center covariates might be present [39, 40]. However, the variable “study center” did not correlate significantly with outcomes and this design made the results more generalizable to daily practice. The large variability in the rate of patients with TBI who underwent SSRF shows that there currently is no consensus on this patient group’s optimal treatment. The retrospective nature of this study might have resulted in missing data or underreporting, but the rate of missing data was < 4% for all variables except BMI and smoking status.

In conclusion, SSRF did not impact the number of ventilator-free days in patients with a flail or a non-flail rib fracture pattern and TBI. In patients with TBI and a non-flail fracture pattern, SSRF was associated with a reduced pneumonia risk. In patients with TBI and a flail chest, a shorter ICU LOS was observed in the SSRF group. In addition, SSRF was a safe procedure in both rib fracture groups and did not hamper neurological recovery. The presence of TBI in patients with a specific severe rib fracture pattern that possibly necessitates SSRF, should not be considered a contraindication for this treatment. In the setting of TBI, the decision to perform SSRF should be made by carefully weighing the risks of surgery against the benefits of both pulmonary and overall recovery.

## References

[1] Ziegler DW, Agarwal NN (1994). The morbidity and mortality of rib fractures. J Trauma.

[2] Lafferty PM, Anavian J, Will RE, Cole PA (2011). Operative treatment of chest wall injuries: indications, technique, and outcomes. J Bone Jt Surg Am.

[3] Hofman M, Andruszkow H, Kobbe P, Poeze M, Hildebrand F (2020). Incidence of post-traumatic pneumonia in poly-traumatized patients: identifying the role of traumatic brain injury and chest trauma. Eur J Trauma Emerg Surg.

[4] Michetti CP, Fakhry SM, Brasel K, Martin ND, Teicher EJ, Newcomb A, TRIPP Study Group (2019). Trauma ICU Prevalence Project: the diversity of surgical critical care. Trauma Surg Acute Care Open.

[5] Dehghan N, de Mestral C, McKee MD, Schemitsch EH, Nathens A (2014). Flail chest injuries: a review of outcomes and treatment practices from the National Trauma Data Bank. J Trauma Acute Care Surg.

[6] de Moya M, Nirula R, Biffl W (2017). Rib fixation: who, what, when?. Trauma Surg Acute Care Open.

[7] Kane ED, Jeremitsky E, Pieracci FM, Majercik S, Doben AR (2017). Quantifying and exploring the recent national increase in surgical stabilization of rib fractures. J Trauma Acute Care Surg.

[8] Rockne WY, Grigorian A, Christian A, Nahmias J, Lekawa M, Dolich M, Chin T, Schubl SD (2021). No difference in mortality between level I and level II trauma centers performing surgical stabilization of rib fracture. Am J Surg.

[9] Choi J, Gomez GI, Kaghazchi A, Borghi JA, Spain DA, Forrester JD (2021). Surgical stabilization of rib fracture to mitigate pulmonary complication and mortality: a systematic review and Bayesian meta-analysis. J Am Coll Surg.

[10] Cataneo AJ, Cataneo DC, de Oliveira FH, Arruda KA, El Dib R, de Oliveira Carvalho PE. Surgical versus nonsurgical interventions for flail chest. Cochrane Database Syst Rev. 2015;(7):CD009919. 10.1002/14651858.CD009919.pub2 PMC9189492 26222250

[11] Schuurmans J, Goslings JC, Schepers T (2017). Operative management versus non-operative management of rib fractures in flail chest injuries: a systematic review. Eur J Trauma Emerg Surg.

[12] Pieracci FM, Majercik S, Ali-Osman F, Ang D, Doben A, Edwards JG, French B, Gasparri M, Marasco S, Minshall C (2017). Consensus statement: surgical stabilization of rib fractures rib fracture colloquium clinical practice guidelines. Injury.

[13] Kasotakis G, Hasenboehler EA, Streib EW, Patel N, Patel MB, Alarcon L, Bosarge PL, Love J, Haut ER, Como JJ (2017). Operative fixation of rib fractures after blunt trauma: a practice management guideline from the Eastern Association for the Surgery of Trauma. J Trauma Acute Care Surg.

[14] Wijffels MME, Prins JTH, Perpetua Alvino EJ, Van Lieshout EMM (2020). Operative versus nonoperative treatment of multiple simple rib fractures: a systematic review and meta-analysis. Injury.

[15] Pieracci FM, Leasia K, Bauman Z, Eriksson EA, Lottenberg L, Majercik S, Powell L, Sarani B, Semon G, Thomas B (2020). A multicenter, prospective, controlled clinical trial of surgical stabilization of rib fractures in patients with severe, nonflail fracture patterns (Chest Wall Injury Society NONFLAIL). J Trauma Acute Care Surg.

[16] Fokin AA, Hus N, Wycech J, Rodriguez E, Puente I. Surgical stabilization of rib fractures: indications, techniques, and pitfalls. JBJS Essent Surg Tech. 2020;10(2):e0032:1–21. 10.2106/JBJS.ST.19.00032 PMC7478329 32944413

[17] Pieracci FM, Lin Y, Rodil M, Synder M, Herbert B, Tran DK, Stoval RT, Johnson JL, Biffl WL, Barnett CC (2016). A prospective, controlled clinical evaluation of surgical stabilization of severe rib fractures. J Trauma Acute Care Surg.

[18] Marasco SF, Davies AR, Cooper J, Varma D, Bennett V, Nevill R, Lee G, Bailey M, Fitzgerald M (2013). Prospective randomized controlled trial of operative rib fixation in traumatic flail chest. J Am Coll Surg.

[19] Wijffels MME, Prins JTH, Polinder S, Blokhuis TJ, De Loos ER, Den Boer RH, Flikweert ER, Pull Ter Gunne AF, Ringburg AN, Spanjersberg WR (2019). Early fixation versus conservative therapy of multiple, simple rib fractures (FixCon): protocol for a multicenter randomized controlled trial. World J Emerg Surg.

[20] Liu T, Liu P, Chen JJ, Xie J, Yang F, Liao YL (2019). A randomized controlled trial of surgical rib fixation in polytrauma patients with flail chest. J Surg Res.

[21] Prins JTH, Van Lieshout EMM, Ali-Osman F, Bauman ZM, Caragounis EC, Choi J, Benjamin Christie D, Cole PA, DeVoe WB, Doben AR (2021). Outcome after surgical stabilization of rib fractures versus nonoperative treatment in patients with multiple rib fractures and moderate to severe traumatic brain injury (CWIS-TBI). J Trauma Acute Care Surg.

[22] Beks RB, Peek J, de Jong MB, Wessem KJP, Oner CF, Hietbrink F, Leenen LPH, Groenwold RHH, Houwert RM (2019). Fixation of flail chest or multiple rib fractures: current evidence and how to proceed. A systematic review and meta-analysis. Eur J Trauma Emerg Surg.

[23] Prevention TCoDCa. Pneumonia (Ventilator-associated [VAP] and non-ventilator-associated Pneumonia [PNEU]) Event 2018. https://www.cdc.gov/nhsn/pdfs/pscmanual/6pscvapcurrent.pdf. Accessed 30 Aug 2021.

[24] DuBose J, Inaba K, Okoye O, Demetriades D, Scalea T, O'Connor J, Menaker J, Morales C, Shiflett T, Brown C (2012). Development of posttraumatic empyema in patients with retained hemothorax: results of a prospective, observational AAST study. J Trauma Acute Care Surg.

[25] Leinicke JA, Elmore L, Freeman BD, Colditz GA (2013). Operative management of rib fractures in the setting of flail chest: a systematic review and meta-analysis. Ann Surg.

[26] Swart E, Laratta J, Slobogean G, Mehta S (2017). Operative treatment of rib fractures in flail chest injuries: a meta-analysis and cost-effectiveness analysis. J Orthop Trauma.

[27] Coleman JR, Leasia K, Douglas IS, Hosokawa P, Lawless RA, Moore EE, Pieracci F (2020). Quantifying the expense of deferring surgical stabilization of rib fractures: operative management of rib fractures is associated with significantly lower charges. J Trauma Acute Care Surg.

[28] Qiu M, Shi Z, Xiao J, Zhang X, Ling S, Ling H (2016). Potential benefits of rib fracture fixation in patients with flail chest and multiple non-flail rib fractures. Indian J Surg.

[29] Fitzgerald MT, Ashley DW, Abukhdeir H, Christie DB (2017). Rib fracture fixation in the 65 years and older population: a paradigm shift in management strategy at a level I trauma center. J Trauma Acute Care Surg.

[30] Pieracci FM, Coleman J, Ali-Osman F, Mangram A, Majercik S, White TW, Jeremitsky E, Doben AR (2018). A multicenter evaluation of the optimal timing of surgical stabilization of rib fractures. J Trauma Acute Care Surg.

[31] Chen Zhu R, de Roulet A, Ogami T, Khariton K (2020). Rib fixation in geriatric trauma: mortality benefits for the most vulnerable patients. J Trauma Acute Care Surg.

[32] Otaka S, Aso S, Matsui H, Fushimi K, Yasunaga H. Effectiveness of surgical fixation for rib fractures in relation to its timing: a retrospective Japanese nationwide study. Eur J Trauma Emerg Surg. 2020. 10.1007/s00068-020-01548-1 PMC7673683 33210171

[33] The Brain Trauma Foundation. The American Association of Neurological Surgeons. The Joint Section on Neurotrauma and Critical Care. Glasgow coma scale score. J Neurotrauma. 2000;17(6–7):563–71. 10.1089/neu.2000.17.563 10937902

[34] Teasdale G, Maas A, Lecky F, Manley G, Stocchetti N, Murray G (2014). The Glasgow Coma Scale at 40 years: standing the test of time. Lancet Neurol.

[35] Prins JTH, Wijffels MME, Pieracci FM. What is the optimal timing to perform surgical stabilization of rib fractures? J Thorac Dis. 2021;13(Suppl 1):S13–S25. 10.21037/jtd-21-649 PMC8371546 34447588

[36] Green EA, Guidry C, Harris C, McGrew P, Schroll R, Hussein M, Toraih E, Kolls J, Duchesne J, Taghavi S. Surgical stabilization of traumatic rib fractures is associated with reduced readmissions and increased survival. Surgery. 2021;91(2):361–368. 10.1016/j.surg.2021.05.032 34215437

[37] Pieracci FM, Agarwal S, Doben A, Shiroff A, Lottenberg L, Whitbeck SA, White TW (2018). Indications for surgical stabilization of rib fractures in patients without flail chest: surveyed opinions of members of the Chest Wall Injury Society. Int Orthop.

[38] Perel P, Wasserberg J, Ravi RR, Shakur H, Edwards P, Roberts I (2007). Prognosis following head injury: a survey of doctors from developing and developed countries. J Eval Clin Pract.

[39] Localio AR, Berlin JA, Ten Have TR, Kimmel SE (2001). Adjustments for center in multicenter studies: an overview. Ann Intern Med.

[40] Choi J, Kaghazchi A, Dickerson KL, Tennakoon L, Spain DA, Forrester JD. Heterogeneity in managing rib fractures across non-trauma and level I, II, and III trauma centers. Am J Surg. 2021;222(4):849–854. 10.1016/j.amjsurg.2021.02.013 33612257

